# P-1631. Implementing an Effective Antibiotic Prescribing Feedback Intervention in a Large Primary Care Network with Emphasis on Bacterial Sinusitis

**DOI:** 10.1093/ofid/ofae631.1797

**Published:** 2025-01-29

**Authors:** Alexander Pomakov, Jineane V Venci, Samia H Lopa, Robert J Fortuna, Alexandra Yamshchikov

**Affiliations:** University of Kentucky, Lexington, Kentucky; University of Rochester Medical Center, Rochester, New York; Dept. of Biostatistics & Computational Biology, Rochester, New York; University of Rochester, Penfield, NY, New York; University of Rochester School of Medicine and Dentistry, Rochester, New York

## Abstract

**Background:**

Education and feedback interventions are a key aspect of antibiotic stewardship in outpatient settings. We examined effectiveness of an antibiotic prescribing feedback intervention in a large primary care network with emphasis on bacterial sinusitis.

**Figure 1.**
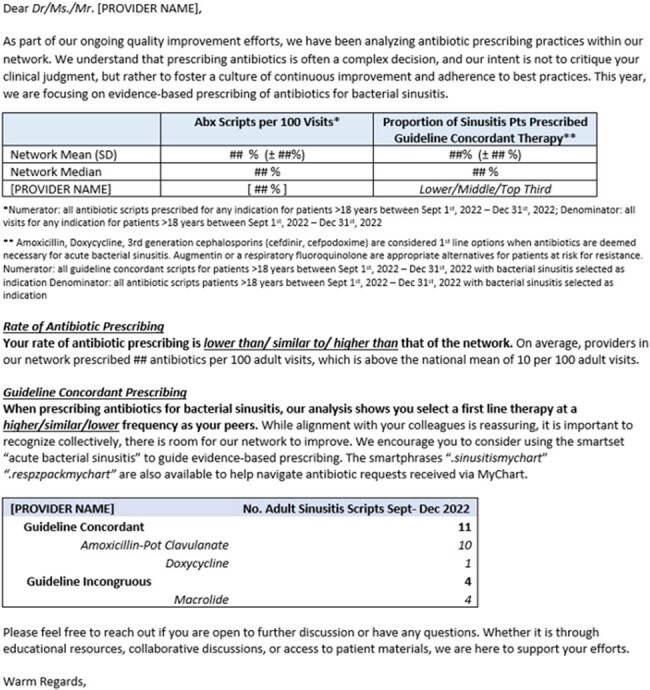
Letter Template for Prescribing and Benchmarking Feedback to Primary Care Network Providers

**Methods:**

We examined prescriptions written by 237 network primary care clinicians between 9/1 -11/31/2022 (baseline n=23048) and 12/1/2023 -2/29/2024 (post intervention n=18885). Feedback was given via individualized letters (Fig 1) comparing providers’ antibiotic prescribing rates and guideline concordant prescribing for sinusitis against network peers and national data. Guideline concordance was defined from local antibiograms and national guidelines. Primary outcomes were rate of antibiotic prescriptions and percentage of guideline concordant sinusitis prescriptions. Data were analyzed in GraphPad and SAS v9.4 with generalized linear mixed model to account for correlation between repeated provider data.

**Table 1.**
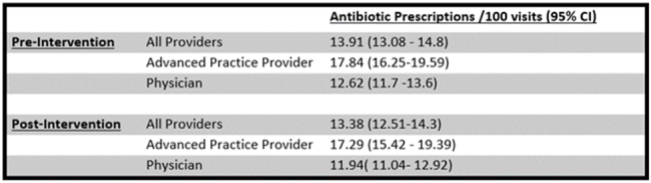
Rates of Antibiotic Prescriptions by Provider Designation Before and After Feedback Intervention

**Results:**

Average rates of antibiotic prescriptions before and after intervention were 13.9 and 13.4/100 visits, a 0.5% absolute reduction and 4.0% relative reduction (p = 0.13). Among providers with baseline >1 standard deviation (SD) antibiotic utilization, average prescribing decreased from 27.7 to 19.7/100 visits (p=0.0002), with increase among those with lowest (> 1 SD) utilization, 5.3 to 7.16/100 visits (p = 0.0048). Physicians’ rates remained lower than non-physician providers (p< 0.0001), and reduction post intervention was similar in both provider groups (p = 0.87, Table 1). Bacterial sinusitis remained most common antibiotic indication (Fig 2). Guideline concordant sinusitis prescriptions pre and post intervention were 69.7% and 83.2% respectively, a 13.5% absolute increase and 19% relative increase (p = < 0.0001) Degree of increase did not differ by provider group (p = 0.76). Antibiotic distribution shifted away from azithromycin, with increases in amoxicillin and doxycycline (Fig 3).

**Figure 2.**
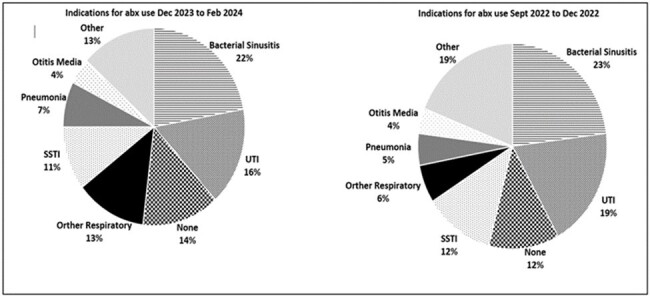
Most Common Indications for Prescribing Antibiotics within URMC Primary Care Network Before and After Feedback Intervention

**Conclusion:**

A prescription feedback intervention in a large primary care network reduced antibiotic prescription rates and non-guideline concordant prescriptions for bacterial sinusitis. The impact of the intervention was most pronounced in prescribers with highest antibiotics utilization pre-intervention.

**Figure 3.**
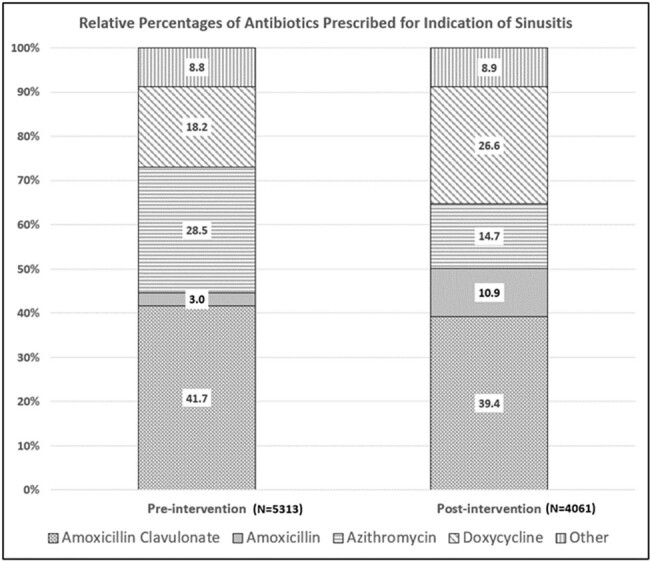
Characterization of Most Common Antibiotics Prescribed by PCN Providers Before and After Feedback Intervention

**Disclosures:**

**All Authors**: No reported disclosures

